# Comparative analysis of the effect of Bioactive Glass 45S5 on enamel erosion progression in human dentitions (in vitro study)

**DOI:** 10.1007/s00784-022-04796-0

**Published:** 2022-11-25

**Authors:** Rehab Samir Salma, Nour Khaled Eldardiry, Haya Ayman Elmaddah, Hoda Ahmed Ismail, Eman M. Salem

**Affiliations:** 1 Faculty of Dentistry, Pediatric and Community Dentistry Department, Pharos University in Alexandria, Sidi Gaber, P.O. Box 37, Alexandria, Egypt; 2 Pharos University in Alexandria, Sidi Gaber, P.O. Box 37, Alexandria, Egypt; 3 Faculty of Dentistry, Oral Biology Department, Pharos University in Alexandria, Sidi Gaber, P.O. Box 37, Alexandria, Egypt

**Keywords:** Bioactive Glass 45S5, Sylc, Dental erosion, Human dentitions, EDX

## Abstract

**Objectives:**

The aim of the present study was to compare the surface morphology alterations, mineral content, and surface roughness of eroded enamel surface versus eroded enamel surface which was preceded by Bioactive Glass 45S5 (BAG45S5) application in both primary and permanent human dentitions.

**Materials and methods:**

Fifty-two primary teeth and fifty-two permanent teeth were selected. Teeth were randomly divided into 4 groups of twenty-six teeth each. Groups A1 and B1 underwent erosion with 1% citric acid, while groups A2 and B2 were subjected to application of BAG45S5 powder followed by the same erosive conditions as A1 and B1. Measurements were performed by scanning electron microscopy (SEM), energy-dispersive X-ray spectroscopy (EDX), and surface profilometry. They were used to examine the surface morphology alterations, mineral content, and surface roughness, respectively.

**Results:**

SEM of enamel which received BAG45S5 showed smoother surface in primary teeth post erosion. EDX analysis showed that enamel exhibited crucial resistance to mineral loss in the group which received BAG45S5 prior to inducing erosion as compared to the induced erosion-only group. This was significant (*p* < 0.005) in both human dentitions. Erosion-only groups showed significantly less surface roughness in permanent teeth (*p* < 0.045). A marked decrease in surface roughness was observed in surfaces receiving BAG45S5, primary teeth (*p* < 0.001), and permanent teeth (*p* < 0.001).

**Conclusions:**

Bioactive Glass 45S5 proved successful against erosive conditions in both primary and permanent teeth with better performance in the permanent teeth so it can be regarded as a means of prevention.

**Clinical relevance:**

Bioactive Glass 45S5 powder could be used not only to remove stains but also as a prophylactic preventive measure against the multiple episodes of acidic food and beverage consumption in children.

## Introduction

Dental erosion is the chronic and progressive, irreversible loss of dental hard tissues caused by a chemical process without any bacterial involvement. It has become a significant clinical challenge in the recent years. This is due to the major lifestyle changes that have led to an increase in the amount and frequency of consumption of acid-containing foods and beverages [1]. Owing to its thinner and less calcified enamel, the primary dentition is more prone to erosion than the permanent dentition. Moreover, primary enamel has a greater inter-prismatic volume fraction and prism-junction density than that of the permanent which in turn result in high porosity and greater rate of mineral diffusion. All of which contribute to the higher liability of primary dentition to erosion [2, 3]. Individuals who are presented with erosion in the primary dentition have an increased risk of developing erosion in the permanent dentition. Thus, early diagnosis and prevention would help in avoiding erosion of the permanent teeth [4].

Erosion could have dramatic effect on the dental tissues and would result in alterations in surface roughness, changes in surface morphology, and even mineral loss of dental tissues [5–7]. These structural changes of dental hard tissues induced by erosive agents can be evaluated by various methods. Erosion could be estimated by measuring surface roughness of enamel. Profilometry and confocal laser scanning microscopy (CLSM) are frequently used to measure tooth surface roughness [8, 9]. Changes in surface hardness, morphology, and mineral composition of dental hard tissues after an acid attack could be also recorded to evaluate dental erosion. Surface morphology alterations can be observed using scanning electron microscopy (SEM), atomic force microscopy (AFM), or CLSM [9–11]. Changes in mineral composition can be detected by energy-dispersive X-ray spectroscopy (EDX), Fourier transform infrared (FTIR), and Raman spectroscopy [12].

Bioactive glasses are biocompatible silicate-based materials, containing calcium and phosphate in an amorphous matrix. They undergo a unique biological reaction at the interface stimulating the formation of a chemical bond between living structures and the material itself [13]. These silicate-based materials are supplied in multiple forms to facilitate manipulation and accommodate for every clinical procedure. Bioactive glass is available as pellets, particulate, powder, mesh, and cones [14]. Their biocompatibility stems from the development of a biologically active hydroxycarbonate apatite layer that bonds to calcified tissues including bone and dental tissues. Bioactive glasses including Bioactive Glass 45S5 (BAG45S5) (calcium-sodium-phospho-silicate compound) is utilized in many dental applications due to its remineralizing and antibacterial effects. It additionally presented better results when used for air polishing and stain removal in comparison to traditional sodium bicarbonate [13].

A study by Dionysopoulos et al. revealed that surface pre-treatment using air abrasion with BAG45S5 may help to prevent enamel surface erosion induced by an acidic drink in bovine teeth [15]. In addition, several other studies in literature were conducted to evaluate the abrasion/erosion processes using bovine teeth as an alternative to human teeth [16–18]. Although bovine teeth were deemed as an acceptable substitute, some voiced their concern regarding the variations of the data from bovine teeth than human teeth due to difference in chemistry and structure. It is crucial to highlight that the bovine enamel is of a higher porosity with bigger crystals than human enamel [19]; therefore, the rate of progression of the demineralizing and remineralizing process is more rapid [16, 20]. This guided the purpose of the current study to overcome the limitations of previous studies to produce more conclusive results, which in turn would reflect more clinical accuracy and can aid in future treatment and preventive programmes. Hence, the aim of this in vitro investigation was to compare the surface morphology alterations, mineral content, and surface roughness of eroded enamel surface versus eroded enamel surface which was preceded by BAG45S5 application in both primary and permanent human dentitions. The null hypothesis was that enamel surface that received BAG45S5 would have a well-remineralized surface layer which can withstand erosive challenge compared to enamel surface subjected to erosion-only in both primary and permanent enamel.

## Materials and methods

The current study is a cross-sectional in vitro study, which was conducted in the Pediatric Dentistry and the Oral Biology Departments, Faculty of Dentistry, Pharos University in Alexandria, Alexandria, Egypt. Ethical approval was obtained from the Research Ethics Committee—Pharos University in Alexandria in adherence to the tenets of the Declaration of Helsinki 1964 and its later amendments (# PUA02202207243036) [21]. Participants were those attending the outpatient clinic during the period October 2021 until December 2021. The purpose of the study was explained to participating individuals and to parents/legal guardians of participating minors who were all allowed to sign a consent form that they are willing to donate their teeth for research purposes.

Sample size was based on 95% confidence level to detect differences in surface roughness and mineral content between eroded enamel surface as opposed to eroded enamel surface preceded by BAG45S5 application. Dionysopoulos et al. reported mean ± SD difference in surface roughness of eroded enamel pre-treated with Bioactive Glass = 0.002 ± 0.001, whereas it was 0.006 ± 0.01 for eroded enamel [22]. The calculated mean ± SD deviation difference =  − 0.004 ± 0.006 and at 95% confidence interval =  − 0.002, 0.01. Therefore, the minimum sample size was calculated to be eleven per group, increased to twelve to make up for laboratory processing errors. The total sample size required = number of groups × number per group = 4 × 12 = 48 [23]. The calculations were performed using MedCalc Statistical Software version 19.0.5.

### Inclusion criteria

Age group 6–20 years; normally shed or serially extracted teeth (permanent teeth, premolars; primary teeth, molars); sound labial/buccal enamel surface.

### Exclusion criteria

Labial/buccal enamel surface with cracks; developmental defects; caries; erosion; fillings.

Fifty-two primary teeth were randomly and equally divided into 2 groups A1 and A2. Fifty-two permanent teeth were also randomly and equally divided into the other 2 groups B1, B2. Randomization was completed using a computer-generated list of random numbers using RAND and RANK functions-Excel (MS Office 365). A serial number was given to each tooth indicating its allocation.

**Fig. 1:**
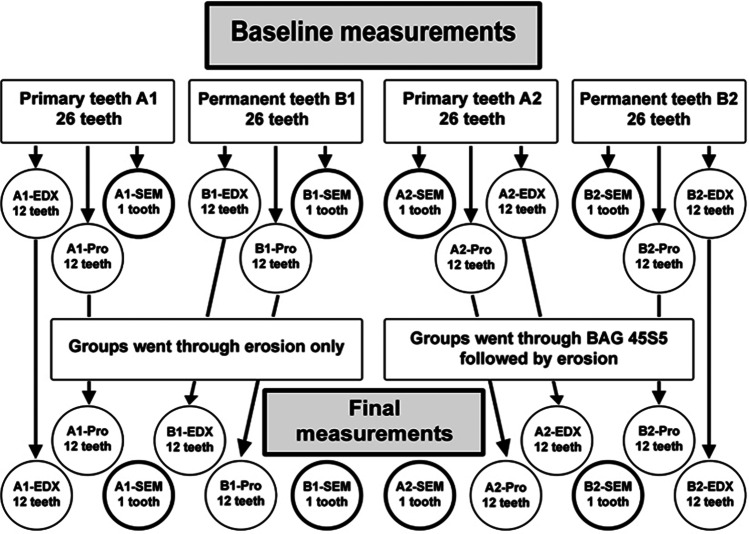
Flowchart of the study

- **Group A1:** twenty-six primary teeth undergoing erosion induction only. Subgroups: two teeth assigned for scanning electron microscopy (SEM) (A1-SEM); twelve teeth for energy-dispersive X-ray spectroscopy (EDX) (A1-EDX); twelve teeth for profilometry (A1-Pro)
- **Group A2:** twenty-six primary teeth receiving BAG45S5 followed by erosion induction. Subgroups: two teeth assigned for SEM (A2-SEM); twelve teeth for EDX (A2-EDX); twelve teeth for profilometry (A2-Pro)
- **Group B1:** twenty-six permanent teeth undergoing erosion induction only. Subgroups: two teeth assigned for SEM (B1-SEM); twelve teeth for EDX (B1-EDX); twelve teeth for profilometry (B1-Pro)
- **Group B2:** twenty-six permanent teeth receiving BAG45S5 followed by erosion induction. Subgroups: two teeth assigned for SEM (B2-SEM); twelve teeth for EDX (B2-EDX); twelve teeth for profilometry (B2-Pro) (Fig. 1)

Collected teeth were kept in 10% formaldehyde solution at 4 °C for 7 days for sterilization [24]. Teeth were rinsed and stored at 4 °C in distilled water with 0.4% sodium azide (to prevent bacterial growth) [20] until completion of the required sample size (54 days). All teeth were cleaned using fluoride-free fine pumice paste; i-Faste (i-dental Innovative Dental Products, Siauliai, Lithuania) to remove any residual organic substances on the enamel surface. Baseline surface morphology alterations, mineral content, and surface roughness for all groups were recorded. Groups A2 and B2 received BAG45S5 (explained in detail in the following section). All teeth in all groups underwent erosion induction (explained in detail in the following section). Post erosion, surface morphology alterations, mineral content, and surface roughness for all groups were recorded.

Bioactive Glass 45S5 **(**BAG45S5) in the form of Sylc® powder (Velopex, Harlesden, UK) with particle size ranging from 25 to 120 µm (Table 1) was applied on the enamel surface in groups A2 and B2. This was done through the air abrasion system, Aquacare® (Velopex, Harlesden, UK) at an air pressure range of 40–46 psi or approximately 2.8–3.2 bar. The device was set to a minimum powder flow and powder uptake. But if increased effect is required, flow and powder settings were increased accordingly. Aquacare® system is a replaceable cartridge system which has 2 modes: air abrasion and air polishing modes. Sylc powder that was used in the current study works with the air polishing mode.

**Table 1:** Composition of the materials used

Product	Composition
Artificial saliva	0.166 g/l of CaCl_2_, 0.059 g/l MgCl_2_, 0.326 g/l KH_2_PO_4_, 0.804 g/l K_2_HPO_4_, 0.625 g/l KCl. 200 g methyl paraben, 10 g sodium carboxymethyl cellulose and distilled water H_2_O. pH = 7
Sylc	Calcium sodium phosphosilicate; 100% NovaMin, particle size: 30–60-90 μm, SiO_2_: 45%, CaO: 24.4%, Na_2_O: 24.6%, P_2_O_5_: 6%
Erosive agent	1% concentration (1 mol/L) citric acid C_6_H_8_O_7_

**Fig. 2:**
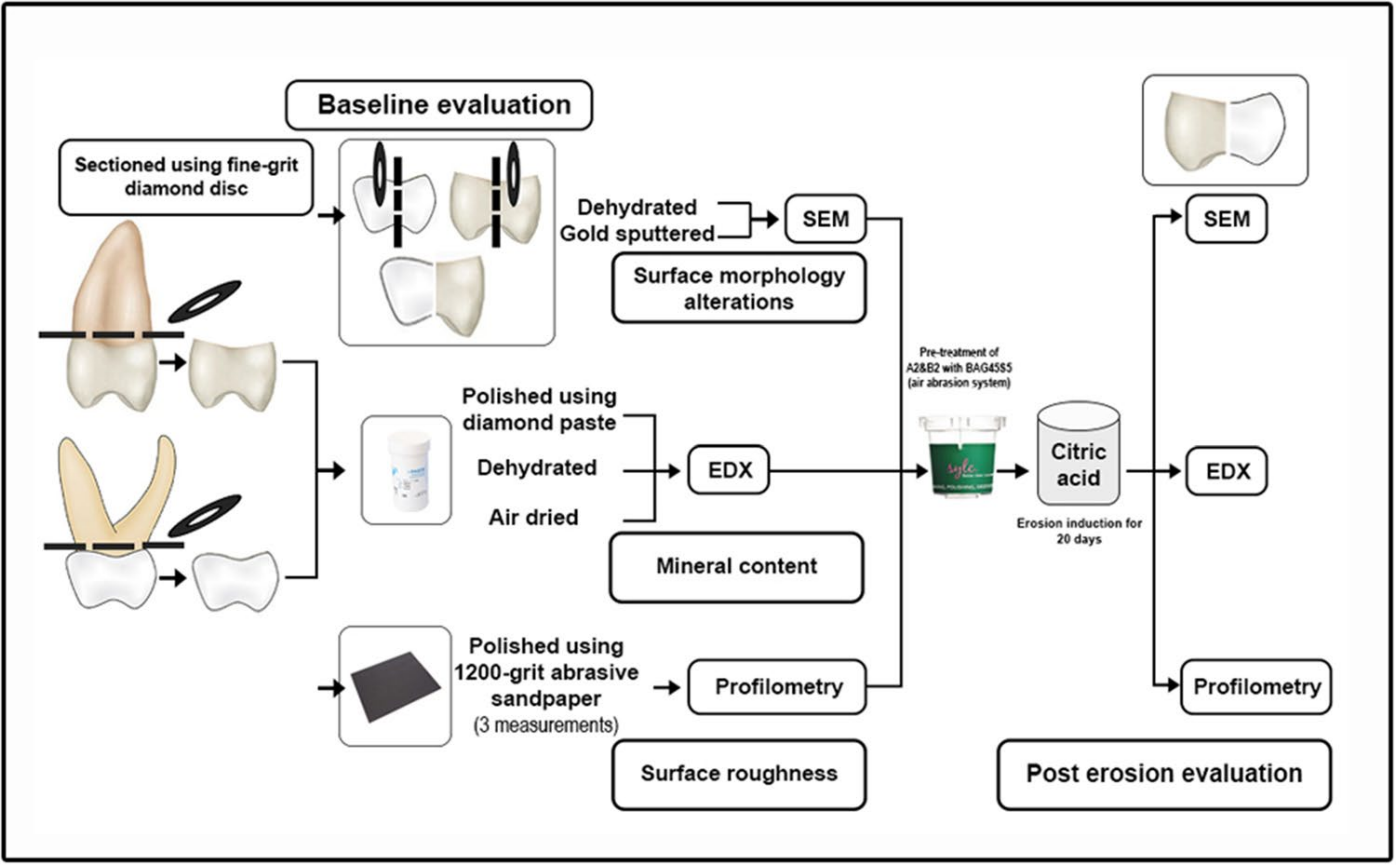
Schematic illustration of the study

Six erosion cycles per day for 20 consecutive days were performed as follows: Every tooth was coated with an acid-resistant nail varnish with the exception of 3 × 2 mm^2^ of exposed area; each tooth was immersed in 20 ml of 1% citric acid (pH 3.2) for 3 min, rinsed with distilled water, and stored in artificial saliva (storage medium) for 65–80 min between the 6 cycles for remineralization (Table 1). By the end of 6 erosion cycles, teeth were re-put overnight in artificial saliva until the start of the following day’s cycles; the previous steps were repeated every day until the completion of 20 days [20, 25, 26]. (Fig. 2)

### Study outcomes

The outcomes of the current study included:

i. Ultra-structure surface morphology alterations
ii. Mineral content (calcium, phosphorous, oxygen, carbon)
iii. Surface roughness

### Outcomes assessment

Assessment of outcomes was performed at baseline and post erosion for all groups.

#### Assessment of ultra-structure surface morphology alterations

Scanning electron microscopy (SEM) JSM-IT200 InTouchScope™ Scanning Electron Microscope (JSM-IT200 Series, JEOL; Tokyo, Japan) was used for this purpose. Two teeth from each group were sectioned into buccal and lingual specimens using a double-sided fine grit water-cooled diamond disc yielding 4 specimens. One specimen of each tooth was randomly selected for baseline assessment of ultra-structure surface morphology alterations, while the other one was used for post erosion assessment. All specimens were dehydrated at the time of evaluation by passed through series of 50%, 70%, and 95% ethyl alcohol for 10 min each and then in absolute alcohol for two changes of 1-h period each. This was followed by drying in vacuum desiccator for 1 h [27]. The assigned specimens were then sputtered with gold to form a fine coat around the surface in a vacuum evaporator (JFC-1100E, JEOL; Tokyo, Japan). Two micrographs were captured for each specimen.

#### Assessment of mineral content

Energy-dispersive X-ray spectroscopy (EDX) JSM-IT200 InTouchScope™ Scanning Electron Microscope (JSM-IT200 Series, JEOL; Tokyo, Japan) was used for this purpose. Assigned teeth were well-polished using diamond paste (1-μm size). Then, they were washed out under running water, dehydrated, and air dried [27]. After drying, the twelve teeth from each group were assessed for their mineral content at baseline as well as post erosion.

#### Assessment of surface roughness

Profilometry using linear scanning stylus; MarSurf PS 10 (Mahr GmbH, Gӧttingen, Germany) ISO 16610–21, Lt = 1.5 mm (0.25 × 5), parameters Ra and Rz was used for this purpose. Twelve teeth from each group were assessed for their surface roughness at baseline and then post erosion. Teeth were firstly polished using 1200-grit abrasive sandpaper to smoothen out any irregularities on the enamel surface. Then, they were stored in artificial saliva for 1 h. For each tooth, three measurements were taken, and an average reading for those three measurements was obtained.

### Statistical analysis

Data were analysed using IBM SPSS software package version 20.0*.* (Armonk, NY: IBM Corp). Qualitative data were described using number and percent. The Kolmogorov–Smirnov test was used to verify the normality of distribution. Quantitative data were described using range mean, standard deviation. Significance of the obtained results was judged at the 5% level. The used tests: Student t-test for normally distributed quantitative variables to compare between two groups and paired t-test for normally distributed quantitative variables to compare between two periods.

## Results

### SEM interpretation (ultra-structure surface morphology alterations)

#### Erosion-only groups

**Fig. 3:**
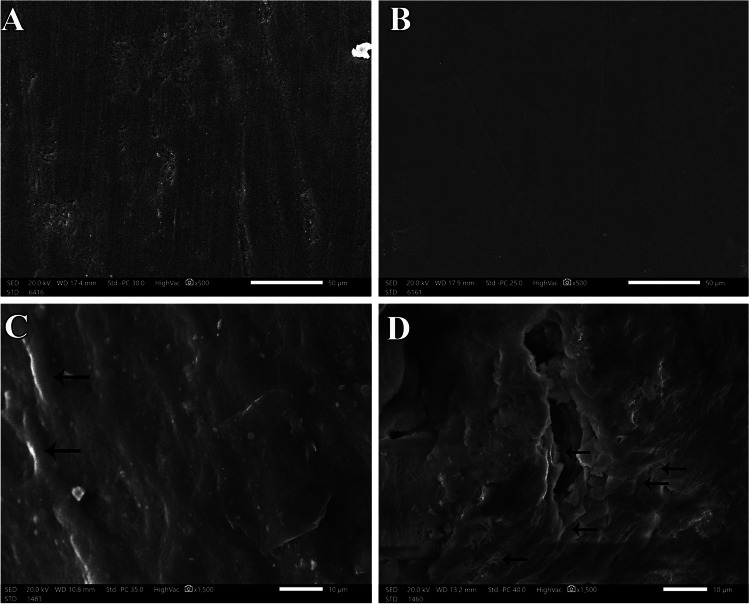
Scanning electron micrograph of the enamel surface at baseline and post erosion for “erosion-only” groups in both primary and permanent teeth. **A** Primary teeth baseline A1-SEM (× 500). **B** Permanent teeth baseline B1-SEM (× 500). **C** Primary teeth post erosion A1-SEM (× 1500). **D** Permanent teeth post erosion B1-SEM (× 1500)

Group A1-SEM micrograph demonstrated generalized smooth surface architecture of the enamel surface (× 500) (Fig. 3A). Group B1-SEM micrograph exhibited intact smooth surface topography and low surface roughness (× 500) (Fig. 3B). Group A1-SEM micrograph demonstrated generalized surface depressions, craters, and erosive material over the enamel surface (× 1500) (Fig. 3C). Group B1-SEM showed generalized surface irregularities of demineralized enamel and wide and deep demineralization porosity with disintegration of the outer enamel layer (× 1500) (Fig. 3D).

#### Pre-treated groups

**Fig. 4:**
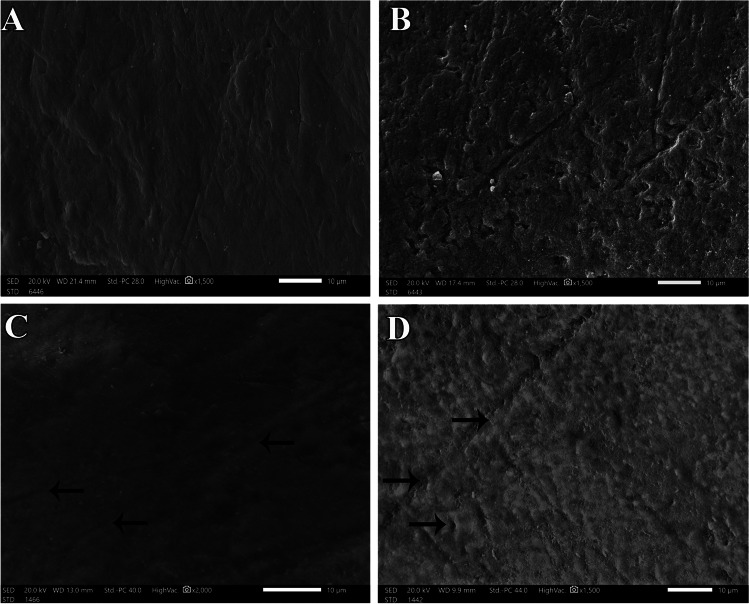
Scanning electron micrograph of the enamel surface at baseline and post erosion for “pre-treated” groups in both primary and permanent teeth. **A** Primary teeth baseline A2-SEM (× 1500). **B** Permanent teeth baseline B2-SEM (× 1500). **C** Primary teeth post erosion A2-SEM (× 2000). **D** Permanent teeth post erosion B2-SEM (× 1500)

Group A2-SEM micrograph was showing microscopic roughness and irregularities in some areas on the enamel surface (× 1500) (Fig. 4A). Group B2-SEM micrograph also was showing microscopic roughness and irregularities on the enamel surface (× 1500) (Fig. 4B). Group A2-SEM demonstrated slight roughness in the enamel surface but with scattered smooth areas. BAG45S5 particles were also observed on enamel covering the surface (× 2000) (Fig. 4C). Group B2-SEM showed particulates of BAG45S5 on a slightly roughened enamel surface with aggregate mass composed of many smaller nanoparticles of the material (× 1500) (Fig. 4D). The surface architecture of the enamel exhibited no evidence of wide-eroded demineralized layer in both A2-SEM and B2-SEM groups but rather a smoother surface was noticed. This was more evident in group A2-SEM than group B2-SEM.

### EDX analysis (mineral content)

#### Group A1-EDX

The (mean ± SD) was at baseline for calcium (Ca) and phosphorus (P) = 24.29 ± 1.24 and 14.30 ± 0.81 respectively which decreased post erosion to = 3.20 ± 1.99 and 1.43 ± 0.88 for Ca and P, respectively. This was statistically significant (*p* < 0.001). Carbon (C) (mean ± SD) showed an increase post erosion = 51.48 ± 2.38 than baseline = 12.15 ± 2.65 which was statistically significant (*p* < 0.001). Oxygen (O_2_) (mean ± SD) showed a decrease post erosion = 43.47 ± 1.54 than baseline = 48.60 ± 1.75 which was statistically significant (*p* < 0.001). Fluoride (F) (mean ± SD) showed a negligible amount at baseline and post erosion with no significant difference (*p* = 0.275).

#### Group B1-EDX

**Fig. 5:**
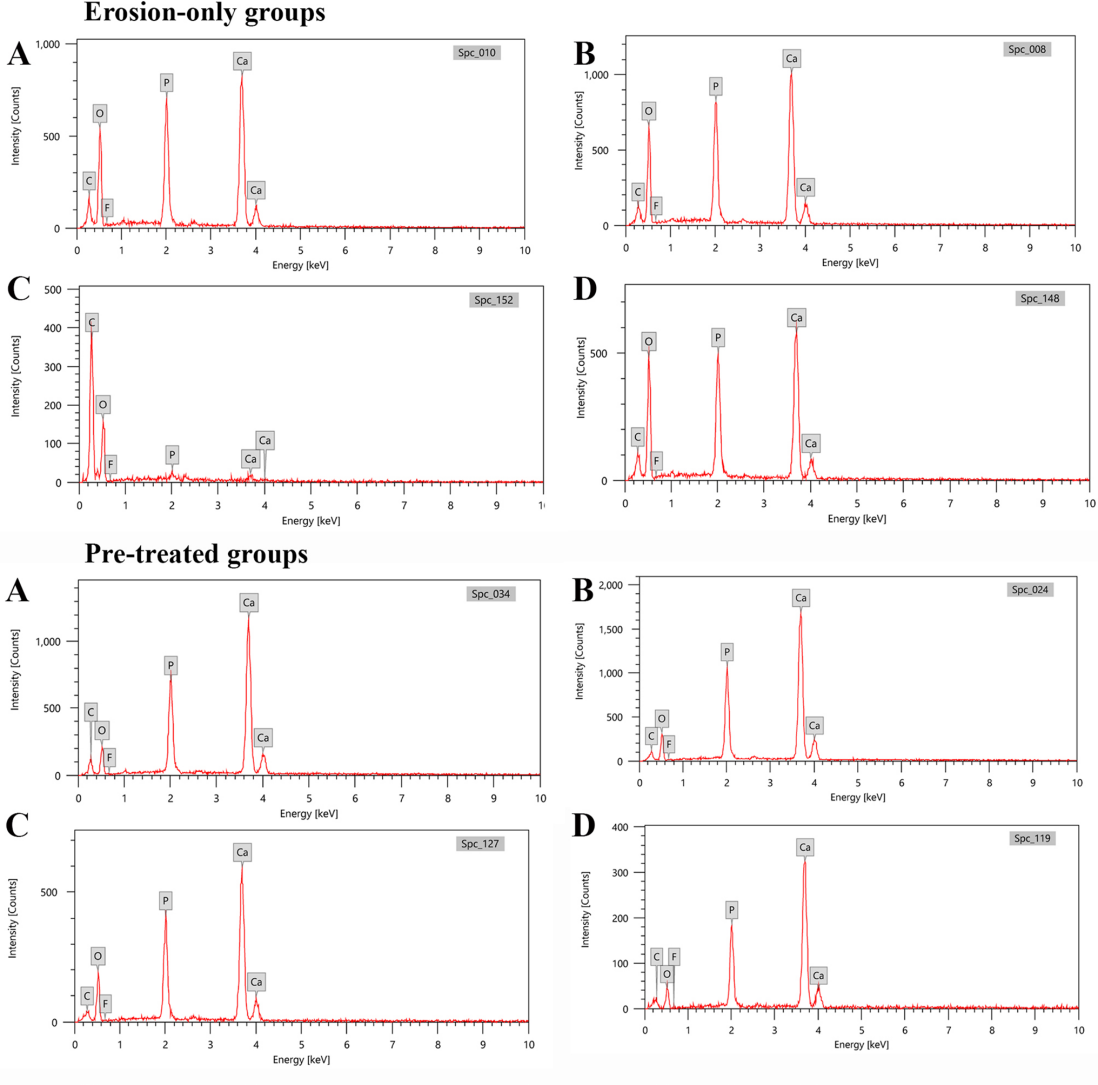
EDX spectra of mineral content at baseline and post erosion for erosion-only groups and for pre-treated groups. Elements are presented by weight (wt%). **A** Primary teeth baseline, **B** primary teeth post erosion, **C** permanent teeth baseline, **D** permanent teeth post erosion

**Fig. 6:**
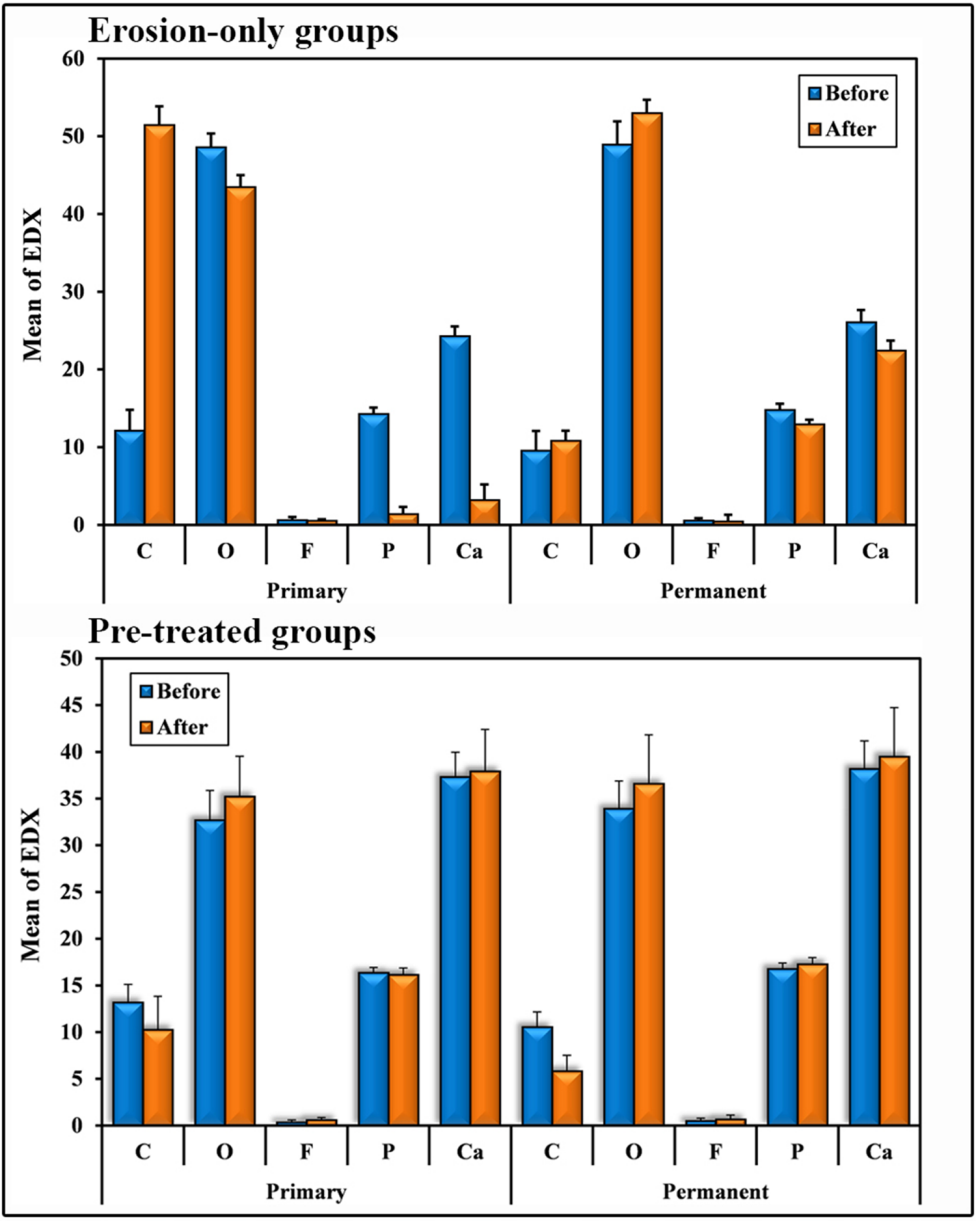
Graph showing comparison between mineral content at baseline and post erosion using EDX for erosion-only groups and for pre-treated groups

The (mean ± SD) was at baseline for Ca and P = 26.09 ± 1.57 and 14.82 ± 0.77 respectively which decreased post erosion to = 22.45 ± 1.27 and 12.95 ± 0.57 for Ca and P, respectively. This was statistically significant (*p* < 0.001). C (mean ± SD) showed an increase post erosion = 10.85 ± 1.26 than baseline = 9.59 ± 2.50 which was statistically non-significant (*p* = 0.182). O_2_ (mean ± SD) showed an increase post erosion = 53.01 ± 1.68 than baseline = 48.96 ± 2.97 which was statistically significant (*p* < 0.001). Fluoride (F) (mean ± SD) showed a negligible amount at baseline and post erosion with no significant difference (*p* = 0.156) (Table 2, Fig. 5, and Fig. 6).

**Table 2:** Comparison between mineral content at baseline and post erosion using EDX in A1-EDX and B1-EDX (“erosion-only” groups)

EDX	Baseline (*n* = 12)	Post erosion (*n* = 12)	*t*	*p*
A1-EDX group (primary)				
C	12.15 ± 2.65	51.48 ± 2.38	62.788^*^	< 0.001^*^
O_2_	48.60 ± 1.75	43.47 ± 1.54	8.818^*^	< 0.001^*^
F	0.65 ± 0.36	0.54 ± 0.18	1.149	0.275
P	14.30 ± 0.81	1.43 ± 0.88	94.107^*^	< 0.001^*^
Ca	24.29 ± 1.24	3.20 ± 1.99	63.333^*^	< 0.001^*^
B1-EDX group (permanent)				
C	9.59 ± 2.50	10.85 ± 1.26	1.425	0.182
O_2_	48.96 ± 2.97	53.01 ± 1.68	4.782^*^	0.001^*^
F	0.59 ± 0.27	0.48 ± 0.81	1.524	0.156
P	14.82 ± 0.77	12.95 ± 0.57	12.151^*^	< 0.001^*^
Ca	26.09 ± 1.57	22.45 ± 1.27	12.800	< 0.001^*^

t: Paired t-test

*p*: *p* value for comparing between baseline and post “erosion-only”

^*^: Statistically significant at *p* ≤ 0.05

#### Group A2-EDX

The (mean ± SD) was comparable for Ca between baseline and post erosion = 37.32 ± 2.65 and 37.92 ± 4.48, respectively. This was non-significant (*p* = 0.335). Also, for P, (mean ± SD) was comparable between baseline and post erosion = 16.36 ± 0.56 and 16.15 ± 0.72, respectively. This was non-significant (*p* = 0.520). C (mean ± SD) showed a decrease post erosion = 10.27 ± 3.56 than baseline = 13.18 ± 1.92 which was non-significant (*p* = 0.085). O_2_ (mean ± SD) showed an increase post erosion = 35.23 ± 4.30 than baseline = 32.69 ± 3.17 which was non-significant (*p* = 0.067). F (mean ± SD) showed an increase post erosion than baseline = 0.61 ± 0.26 and = 0.37 ± 0.22, respectively. This was statistically significant (*p* = 0.004).

#### Group B2-EDX

The (mean ± SD) was comparable for Ca between baseline and post erosion = 38.19 ± 2.99 and 39.49 ± 5.26, respectively. This was non-significant (*p* = 0.255). Also, for P, (mean ± SD) was comparable between baseline and post erosion = 16.78 ± 0.61 and 17.29 ± 0.70, respectively. This was non-significant (*p* = 0.064). C (mean ± SD) showed a decrease post erosion = 5.83 ± 1.68 than baseline = 10.55 ± 1.63 which was statistically significant (*p* < 0.001). O_2_ (mean ± SD) showed an increase post erosion = 36.59 ± 5.24 than baseline = 33.93 ± 2.94 which was statistically significant (*p* = 0.049). F (mean ± SD) showed a negligible amount at baseline and post erosion with no significant difference (*p* = 0.352) (Table 3, Fig. 5, and Fig. 6).

**Table 3:** Comparison between mineral content at baseline and post erosion using EDX in A2-EDX and B2-EDX (pre-treated groups)

EDX	Baseline (*n* = 12)	Post erosion (*n* = 12)	*t*	*p*
A2-EDX group (primary)				
C	13.18 ± 1.92	10.27 ± 3.56	1.895	0.085
O_2_	32.69 ± 3.17	35.23 ± 4.30	2.029	0.067
F	0.37 ± 0.22	0.61 ± 0.26	3.564^*^	0.004^*^
P	16.36 ± 0.56	16.15 ± 0.72	0.665	0.520
Ca	37.32 ± 2.65	37.92 ± 4.48	1.008	0.335
B2-EDX group (permanent)				
C	10.55 ± 1.63	5.83 ± 1.68	8.011^*^	< 0.001^*^
O_2_	33.93 ± 2.94	36.59 ± 5.24	2.207^*^	0.049^*^
F	0.50 ± 0.30	0.67 ± 0.45	0.971	0.352
P	16.78 ± 0.61	17.29 ± 0.70	2.059	0.064
Ca	38.19 ± 2.99	39.49 ± 5.26	1.201	0.255

t: Paired t-test

*p*: *p* value for comparing between baseline and post erosion

^*^: Statistically significant at *p* ≤ 0.05

#### A1-EDX and B1-EDX groups

Post erosion, the (mean ± SD) of Ca content change decreased in A1-EDX than that of B1-EDX =  − 21.09 ± 1.15 and − 3.64 ± 0.98, respectively. This change was statistically significant (*p* < 0.001). The (mean ± SD) of P content change decreased in A1-EDX than that of B1-EDX =  − 12.87 ± 0.47 and − 1.87 ± 0.53, respectively. This change was statistically significant (*p* < 0.001). The (mean ± SD) of O_2_ content change decreased in A1-EDX than that of B1-EDX =  − 5.13 ± 2.01 and 4.06 ± 2.94, respectively. This change was statistically significant (*p* < 0.001). The (mean ± SD) of F content change decreased in A1-EDX than that of B1-EDX =  − 0.11 ± 0.34 and 0.20 ± 0.45, respectively. This change was non-significant (*p* = 0.115). The (mean ± SD) of C content change increased in A1-EDX than that of B1-EDX = 39.33 ± 2.17 and 1.25 ± 3.05, respectively. This change was statistically significant (*p* < 0.001).

#### A2-EDX and B2-EDX groups

**Fig. 7:**
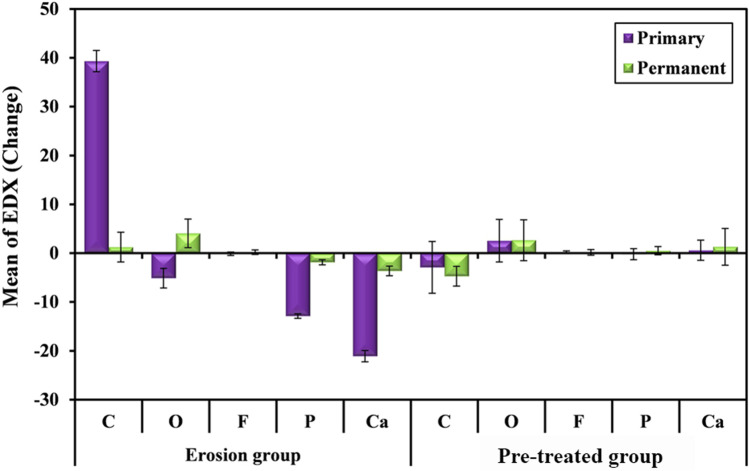
Graph showing comparison of the mineral content change between primary and permanent teeth post erosion using EDX

Post erosion, the (mean ± SD) of Ca content change decreased in A2-EDX than that of B2-EDX = 0.60 ± 2.06 and 1.31 ± 3.76, respectively. This change was non-significant (*p* = 0.476). The (mean ± SD) of P content change decreased in A1-EDX than that of B1-EDX =  − 0.22 ± 1.14 and 0.51 ± 0.86, respectively. This change was non-significant (*p* = 0.159). The (mean ± SD) of O_2_ content change showed comparable change in A1-EDX and B1-EDX = 2.55 ± 4.35 and 2.66 ± 4.18, respectively. This change was non-significant (*p* = 0.947). The (mean ± SD) of F content showed comparable change in A1-EDX and B1-EDX = 0.24 ± 0.23 and 0.16 ± 0.59, respectively. This change was non-significant (*p* = 0.716). The (mean ± SD) of C content change increased in A1-EDX than that of B1-EDX =  − 2.90 ± 5.31 and − 4.72 ± 2.04, respectively. This change was non-significant (*p* = 0.340) (Table 4 and Fig. 7).

**Table 4:** Comparison of the mineral content change between primary and permanent teeth post erosion using EDX

Change between groups post erosion	Primary (*n* = 12)	Permanent (*n* = 12)	t	p
Post erosion in A1-EDX and B1-EDX groups				
C	39.33 ± 2.17	1.25 ± 3.05	36.948^*^	< 0.001^*^
O_2_	− 5.13 ± 2.01	4.06 ± 2.94	11.666^*^	< 0.001^*^
F	− 0.11 ± 0.34	0.20 ± 0.45	1.710	0.115
P	− 12.87 ± 0.47	− 1.87 ± 0.53	59.753^*^	< 0.001^*^
Ca	− 21.09 ± 1.15	− 3.64 ± 0.98	44.869^*^	< 0.001^*^
Post erosion in A2-EDX and B2-EDX groups				
C	− 2.90 ± 5.31	− 4.72 ± 2.04	0.997	0.340
O_2_	2.55 ± 4.35	2.66 ± 4.18	0.069	0.947
F	0.24 ± 0.23	0.16 ± 0.59	0.374	0.716
P	− 0.22 ± 1.14	0.51 ± 0.86	1.512	0.159
Ca	0.60 ± 2.06	1.31 ± 3.76	0.737	0.476

t: Paired t-test

*p*: *p* value for comparing between primary and permanent teeth

^*^: Statistically significant at *p* ≤ 0.05

#### A1-EDX and A2-EDX

The mineral content change of Ca and P significantly increased in A2-EDX than A1-EDX as Ca = 37.92 ± 4.48 and 3.20 ± 1.99 respectively (p < 0.001) and *P* = 16.15 ± 0.72 and 1.43 ± 0.88 respectively (*p* < 0.001). The mineral content change of C and O_2_ significantly decreased in A2-EDX than A1-EDX as *C* = 10.27 ± 3.56 and 51.48 ± 2.38 respectively (*p* < 0.001) and O_2_ = 35.23 ± 4.30 and 43.47 ± 1.54 respectively (*p* < 0.001). F content change was comparable between groups A2-EDX and A1-EDX = 0.61 ± 0.26 and 0.54 ± 0.18 respectively which was non-significant (*p* = 0.454).

#### B1-EDX and B2-EDX

The mineral content change of Ca and P significantly increased in B2-EDX than B1-EDX as Ca = 39.49 ± 5.26 and 22.45 ± 1.27 respectively (*p* < 0.001), and *P* = 17.29 ± 0.70 and 12.95 ± 0.57 respectively (*p* < 0.001).

**Fig. 8:**
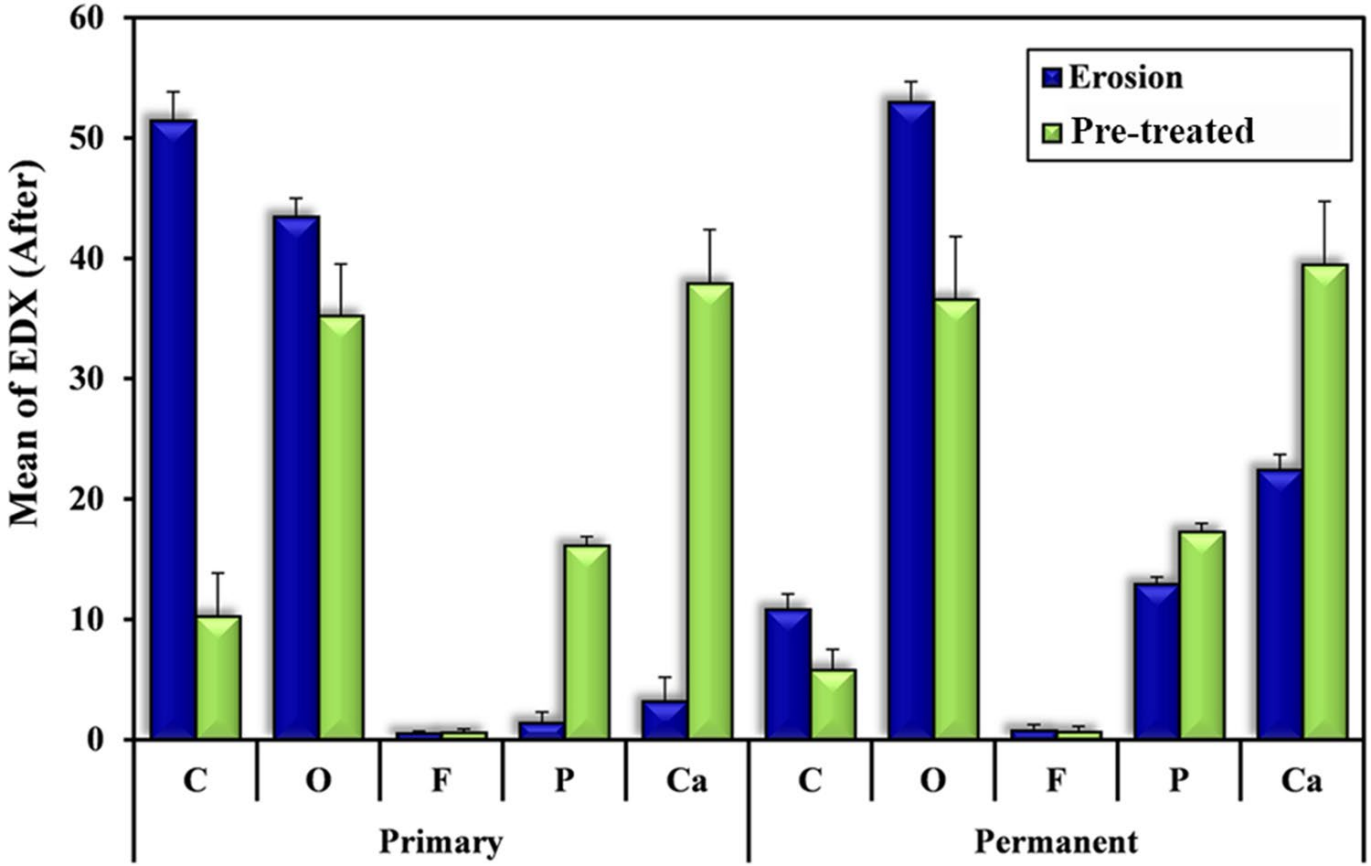
Graph showing comparison of the mineral content change between “erosion-only” and “pre-treated” groups using EDX

The mineral content change of C and O_2_ significantly decreased in B2-EDX than B1-EDX as C = 5.83 ± 1.68 and 10.85 ± 1.26 respectively (*p* < 0.001) and O_2_ = 36.59 ± 5.24 and 53.01 ± 1.68 respectively (*p* < 0.001). F content change was comparable between groups B2-EDX and B1-EDX = 0.67 ± 0.45 and 0.79 ± 0.48 respectively which was non-significant (*p* = 0.537) (Table 5 and Fig. 8).

**Table 5:** Comparison of the mineral content change between “erosion-only” and “pre-treated” groups using EDX

Change in EDX post erosion	Erosion-only (*n* = 12)	Pre-treated (*n* = 12)	t	p
A1-EDX and A2-EDX groups (primary)				
C	51.48 ± 2.38	10.27 ± 3.56	33.334^*^	< 0.001^*^
O_2_	43.47 ± 1.54	35.23 ± 4.30	6.240^*^	< 0.001^*^
F	0.54 ± 0.18	0.61 ± 0.26	0.762	0.454
P	1.43 ± 0.88	16.15 ± 0.72	44.886^*^	< 0.001^*^
Ca	3.20 ± 1.99	37.92 ± 4.48	24.527^*^	< 0.001^*^
B1-EDX and B2-EDX groups (permanent)				
C	10.85 ± 1.26	5.83 ± 1.68	8.276^*^	< 0.001^*^
O_2_	53.01 ± 1.68	36.59 ± 5.24	10.340^*^	< 0.001^*^
F	0.79 ± 0.48	0.67 ± 0.45	0.627	0.537
P	12.95 ± 0.57	17.29 ± 0.70	16.640^*^	< 0.001^*^
Ca	22.45 ± 1.27	39.49 ± 5.26	10.918^*^	< 0.001^*^

t: Paired t-test

*p*: *p* value for comparing between erosion-only and pre-treated groups

^*^: Statistically significant at *p* ≤ 0.05

### Profilometry assessment (surface roughness)

**Fig. 9:**
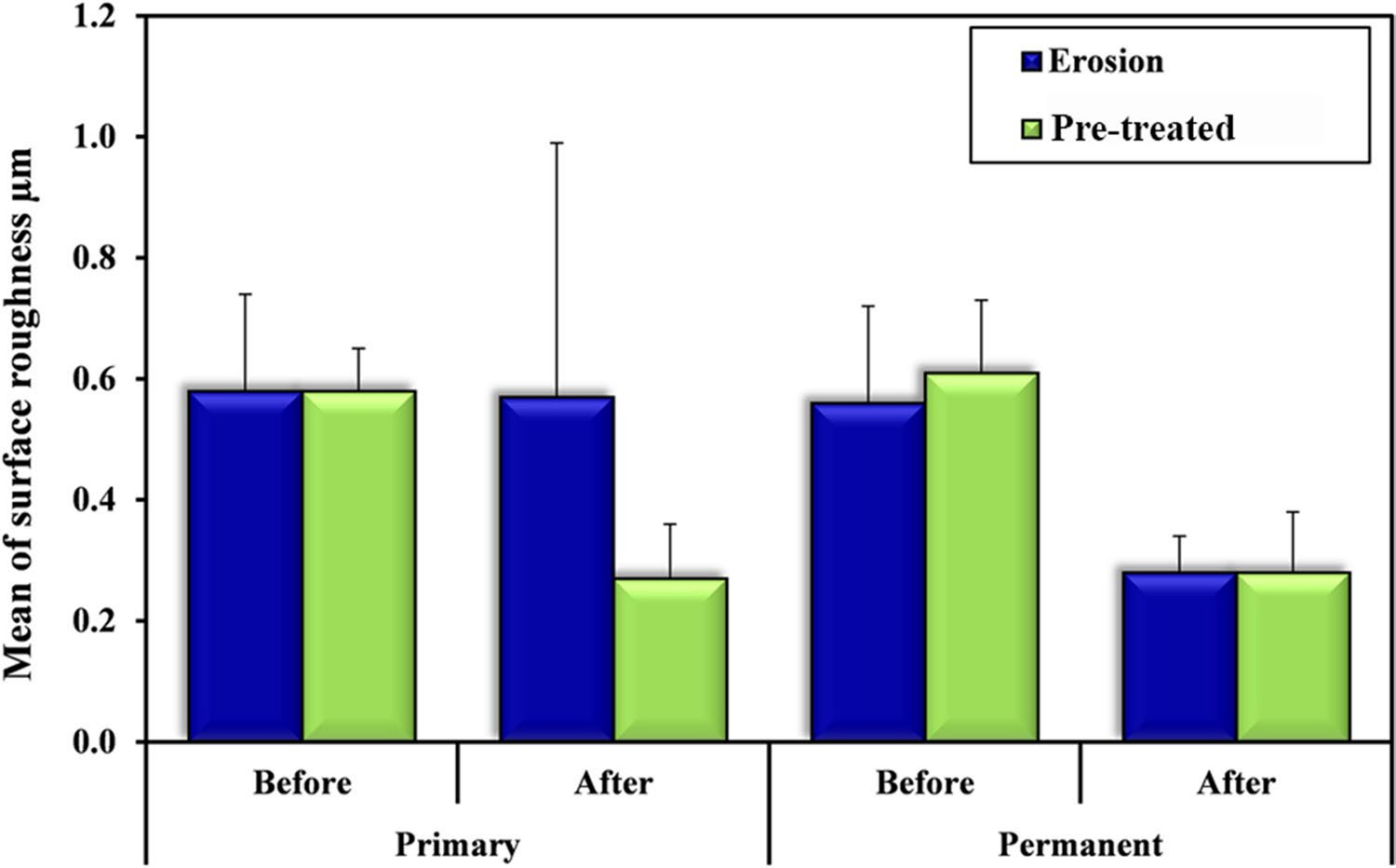
Graph showing comparison between “erosion-only” and “pre-treated” groups according to surface roughness

(Mean ± SD) of A1-Pro readings at baseline was = 0.58 ± 0.16, while for post erosion, it was = 0.57 ± 0.42 which was not statistically significant (*p*_0_ = 0.955). (Mean ± SD) of B1-Pro readings at baseline was = 0.56 ± 0.16, while for post erosion, it was decreased to = 0.28 ± 0.06, and this was statistically significant (*p*_0_ < 0.001). (Mean ± SD) of A2-Pro readings at baseline was = 0.58 ± 0.07, while for post erosion, it was decreased to = 0.27 ± 0.09, and this was statistically significant (*p*_0_ < 0.001). (Mean ± SD) of B2-Pro readings at baseline was = 0.61 ± 0.12, while for post erosion, it was decreased to = 0.28 ± 0.10, and this was statistically significant (*p*_0_ < 0.001). The change in surface roughness from baseline to post erosion significantly decreased in B1-Pro than that of A1-Pro = 0.56 ± 0.16/0.28 ± 0.06 and = 0.58 ± 0.16/0.57 ± 0.42 respectively (*p*_1_ = 0.045). The change in surface roughness from baseline to post erosion was similarly decreased in A2-Pro and B2-Pro = 0.58 ± 0.07/0.27 ± 0.09 and = 0.61 ± 0.12/0.28 ± 0.10 respectively (*p*_1_ = 0.563). The change in surface roughness, post erosion, between group A1-Pro = 0.57 ± 0.42, and A2-Pro = 0.27 ± 0.09 was significantly less in group A2-Pro (*p* = 0.026). The change in surface roughness, post erosion, between group B1-Pro = 0.28 ± 0.06, and B2-Pro = 0.28 ± 0.10 was not significant (*p* = 0.960) (Table 6 and Fig. 9).

**Table 6:** Comparison between “erosion-only” and “pre-treated” groups according to surface roughness

Surface roughness µm		Erosion-only A1-Pro (*n* = 12) B1-Pro (*n* = 12)	Pre-treated A2-Pro (*n* = 12) B2-Pro (*n* = 12)	t	p
Primary A1-Pro, A2-Pro	**Baseline**	**(*n* = 12)**	**(*n* = 12)**	0.066	0.948
	Mean ± SD	0.58 ± 0.16	0.58 ± 0.07		
	**Post**	**(*n* = 12)**	**(*n* = 12)**	2.385^*^	0.026^*^
	Mean ± SD	0.57 ± 0.42	0.27 ± 0.09		
	**t**_**0**_** (*p***_**0**_**)**	**0.057(0.955)**	8.865 (< 0.001^*^)		
Permanent B1-Pro, B2-Pro	**Baseline**	**(*n* = 12)**	**(*n* = 12)**	0.760	0.456
	Mean ± SD	0.56 ± 0.16	0.61 ± 0.12		
	**Post**	**(*n* = 12)**	**(*n* = 12)**	0.051	0.960
	Mean ± SD	0.28 ± 0.06	0.28 ± 0.10		
	**t**_**0**_** (*p***_**0**_**)**	5.661 (< 0.001^*^)	7.573 (< 0.001^*^)		
	**t**_**1**_** (*p***_**1**_**)**	2.120 (0.045^*^)	**0.588 (0.563)**		

t: Student t-test

t_0_: Student t-test

t_1_: Paired t-test

*p*: *p* value for comparing between erosion-only and pre-treated groups

*p*_0_: *p* value for comparing between baseline and post erosion in each group

*p*_1_: *p* value for comparing between primary and permanent in each group

^*^: Statistically significant at *p* ≤ 0.05

## Discussion

The present study aimed to provide a minimally invasive available clinical solution to intercept erosion and its consequences by testing Bioactive Glass 45S5 (BAG45S5) on both human primary and permanent dentitions. To overcome the lack of reliability in the use of bovine teeth instead of human ones, teeth were used from both human dentitions instead of bovine teeth for more favourable clinically applicable results. In a review of literature conducted by Yassen et al. [28] and based on their findings of a large number of studies on either bovine or human teeth, they concluded that inconsistent data existed regarding whether bovine teeth can be considered appropriate substitute for human teeth or not and that interpretation of the results is much dependent on knowing the morphological, chemical composition, and physical property differences between human and bovine teeth. Although Attin et al. [16] stated that the use of bovine could be considered a perfect substitute to human teeth, White et al. [17] concluded that the rate of erosion progression in bovine teeth was 30% faster than that of human teeth.

Dissolution of the enamel usually occurs when it is exposed to undersaturated solutions at a pH less than the critical pH, which is thought to be 5.5 [29]. Therefore, the employed erosive cycle protocol in the study was determined to be prolonged exposure of enamel to a critical pH below 5.5, at which enamel would demineralize and soften [26]. One percent citric acid solution was used as an erosive agent. This was applied instead of using artificial juices, carbonated drinks, and fresh fruit drinks to maintain a consistent pH throughout the experiment and to avoid any possible discrepancies within the results due to variations of the acidic solution [30, 31]. The concentration selected was used to mimic the pH of organic citric acid (3.2) which is typically found in most bottled drinks and fruits. Also, substituting typically used erosive solutions was done to achieve a possible critical pH for the chemical dissolution that is not associated with plaque fluid pH. The exposure time was decided according to the concentrations and the solubility rates of principal mineral constituents of the tooth as well as the pH of the citric acid used [32]. Moreover, the use of artificial saliva throughout the cycle schedule in between the exposure times allowed for mimicking the intrinsic flow of saliva within the oral cavity, to obtain results close to that would occur naturally in a patient’s mouth.

BAG45S5 has been widely studied for its effectiveness on enamel remineralization. It improved the microhardness of the subsurface-eroded enamel surface [33]. A fluoride containing bioactive glass paste showed the formation of crystal-like structures on top of enamel demineralized surfaces [34]. It potentially can remineralize white spot lesions [35, 36]. Additionally, orthodontic brackets that are bonded to pre-treated enamel surface with BAG45S5 showed stable shear bond strength values [36]. BAG45S5 demonstrated slight alteration to the enamel surface roughness when used in removing residual orthodontic adhesive following bracket debonding via air abrasion [37, 38]. Moreover, bioactive glass-containing varnish was found to regain surface microhardness of early carious lesions or demineralized enamel surfaces [39].

Changes in surface roughness commonly occur after the interaction of acidic solutions with tooth structures. While the effect of erosive solutions on the surface roughness depends on the thickness of the aprismatic layer, the non-treated permanent enamel with its thin aprismatic surface layer demonstrated a notable roughness after exposure to the erosive acid, whereas the non-treated primary enamel with its generally uniform and thicker aprismatic layer presenting a more stable surface texture was not as affected by the erosive agent [40]. On the other hand, the enamel receiving BAG45S5 showed smoother surface in both dentitions after applying the erosive agent. The smoother surfaces are believed to be due to the occlusion of the inter-prismatic spaces in enamel by particulates of BAG45S5 [41]. Bakry et al. [42] reported that the formed crystals are gradually transformed into the stable hydroxyapatite crystals after storage in saliva. This was provided in the current study during the erosive cycles as overnight storage for the whole period.

Moreover, the results of the current study revealed that both human dentitions were showing significant loss of calcium and phosphate ions post the erosive challenge. The dissolution was expected to occur due to the weak constitution of the calcium-deficient carbonated hydroxyapatite crystals present in the enamel layer causing a distorted lattice conformation that is more soluble in acidic environments. The primary dentition presented greater mineral loss and surface distortion, because of the higher number of carbonate molecules in the hydroxyapatite crystals in the primary enamel as well as the presence of copious organic content specific to primary enamel [43]. This is demonstrated through the exceptionally high rates of carbon ions post the erosive challenge denoting almost complete dissolution of the enamel layer and consequent exposure of the underlying dentin layer.

On the contrary, enamel surface that was pre-treated by BAG45S5 before the erosive challenge showed decreased rates of carbon ions when compared to the baseline. Moreover, there was no significant change in calcium or phosphate content after erosion also compared to baseline. The effect of BAG45S5 on enamel lessened the effect of erosion on either primary or permanent enamel compared to enamel of erosion-only surfaces. BAG45S5 was suggested to be able to restore early erosive enamel lesions with complete loss of hydroxyapatite crystal content [44]. In the present work, it was employed as protective measure before being subjected to erosive acids. The mechanism controlling its action could be suggested as follows: as air abrasion is started, mix of BAG45S5 powder with ejected water would result in creation of acid resistant calcium phosphate compounds forming a layer of hydroxycarbonate apatite which is structurally similar to biological apatite [42, 45]. This layer acts, upon exposure to acids, as a barrier limiting the effect of citric acid within the enamel. Also, some minerals would dissolve within the erosive solution which would raise the pH and decrease the erosion potential activity [46]. Similarly, Abbassy et al. [30], studying the capability of BAG45S5 of protecting enamel surrounding orthodontic brackets when exposed to erosive challenge, proposed that the formation of an interaction layer could be the reason of resisting development of significant enamel erosive lesion. They explained that mixing BAG45S5 with phosphoric acid would release calcium ions which combine with phosphate ions released from the phosphoric acid forming calcium phosphate compounds that deposit on the enamel surface [44]. Abbassy [31] evaluated the effectiveness of a resin bioactive enamel sealer with BAG45S5 in protecting the enamel adjacent to orthodontic brackets against erosion. This bioactive sealer showed complete coverage of the enamel surface which acted as a protective means against erosion. This was explained by the formation of two protective layers. The first layer was rich in silica and was on top of the glass particles. The second layer was rich in calcium and phosphate which acted as a barrier of acid-resistant crystals on top of the enamel surface.

On demineralized enamel surface, BAG45S5 acted in some way differently as suggested by several studies. Abbassy et al. [36], in their study, used BAG45S5 paste as a remineralizing agent for demineralized enamel and found that it formed a crystalline layer covering the whole treatment area. They suggested that when the BAG45S5 powder was mixed with diluted phosphoric acid that was not in direct contact with the enamel surface, it released calcium and phosphate which penetrated the outer enamel surface and helped in remineralization of the white spot lesion in addition to forming a layer of calcium phosphate salts. They also suggested the formation of soluble silanol compounds which would be washed out by water availability [42, 47].

Throughout the comparison between both dentitions, the permanent dentition which received BAG45S5 showed slightly better response following exposure to induced erosive challenge. Permanent enamel has better consolidation with particles of BAG45S5 due to the intrinsically higher inorganic content as well as morphological differences, indicating that the permanent enamel allows better integration of Bioactive Glass with the inherent calcium and phosphate ions to form more hydroxycarbonate apatite crystals [43]. Since the mechanism of action of BAG45S5 was observed after the interaction of the glass compound with saliva, forming a hydroxycarbonate apatite layer which chemically bonded to enamel, thus, the incorporation of the BAG45S5 particles into the enamel tissues is believed to enhance resistance against solubility by acidic solutions [48]. This assumption was validated through the present study which demonstrated the protective effect of the BAG45S5 against the erosive challenge on the human enamel surface. Hence, the null hypothesis of the study was not rejected.

## Conclusion

It can be concluded that applying Bioactive Glass 45S5 (BAG45S5) to the enamel surface prior to inducing erosion showed better results than inducing erosion alone in both primary and permanent dentitions. Consequently, BAG45S5 can limit or even prevent dental erosion in both primary and permanent dentitions by protecting the enamel surface from acid dissolution, with better performance on the permanent enamel. This means that BAG45S5 could be considered a promising method of erosion prevention. It could be implemented as a part of the preventive program tailored to children who show high consumption of juice and acidic beverages. Further clinical trials on children are needed to assess the effect on teeth sensitivity of using BAG45S5 prior to the repeated episodes of acidic food and beverage intake.

## Study limitations

Being dependant on collecting human teeth, difficulty was encountered in collecting the required number of teeth which led to compensating this through teeth sectioning.

## Data Availability

All data are readily available upon reasonable request.
